# Effect of six weeks of oral *echinacea purpurea* supplementation on nitric oxide production

**DOI:** 10.1186/1550-2783-9-S1-P21

**Published:** 2012-11-19

**Authors:** Tyler D Martin, Michael S Green, Malcolm T Whitehead, Timothy P Scheett, Michael J Webster, Geoffrey M Hudson

**Affiliations:** 1 Department of Kinesiology and Health Promotion, Troy University, Troy, AL, 36082, USA; 2 Department of Physical Therapy, Arkansas State University, State University, AR, 72467, USA; 3 Department of Health and Human Performance, College of Charleston, Charleston, SC, 29424, USA; 4 School of Human Performance and Recreation, The University of Southern Mississippi, Hattiesburg, MS, 39406, USA

## Background

*Echinacea purpurea*, a purple coneflower plant of the compositae family (Asteraceae), is native to North America and commonly used as an herbal supplement to enhance immune function. *Echinacea purpurea* has been shown to stimulate macrophage activity which is a known stimulator of nitric oxide (NO) production. *Echinacea purpurea* supplementation (8,000 mg·d^-1^) in untrained (42.5 ± 1.6 mL·kg^-1^·min^-1^) males was shown to elicit a 63% increase (p < 0.05) in serum erythropoietin (EPO) following two weeks of supplementation. This is supported in part by earlier findings which indicated that four weeks of *Echinacea purpurea* supplementation demonstrated a non-significant increase in maximal oxygen uptake (VO_2max_). It is plausible that Echinacea-induced EPO production may stimulate physiological responses independent of and/or in addition to erythropoiesis. There is also evidence suggesting EPO has vasculo-protective effects including the activation of endothelial nitric oxide synthase (eNOS). Based on these findings, a proposed non-hematological response to the Echinacea-induced increase in EPO could be enhanced NO production. The purpose of this investigation was to determine whether six weeks of oral *Echinacea purpurea* supplementation augmented NO production as a result of an Echinacea-induced increase in EPO and/or Echinacea-induced macrophage activity.

## Methods

Twenty-four males (mean ± SE): age = 25.2 ± 1.4 yr, height = 178.1 ± 1.4 cm, mass = 78.1 ± 1.6 kg, percent body fat = 12.7 ± 0.9 %, VO_2max_ = 52.9 ± 0.9 mL·kg^-1^·min^-1^ were randomly grouped using a matched-pair, double-blind design and self-administered 8,000 mg·d^-1^ (2,000 mg × 4 times·d^-1^) of either *Echinacea purpurea* (ECH) (n=12) or placebo (PLA) (n=12) for 42 consecutive days. Blood samples were collected prior to supplementation (day-0) and every two weeks during the supplementation period (day-14, -28, and -42) and were analyzed for nitrite and total nitrite (nitrite/nitrate) concentrations. Separate 2 × 4 (Group × Time) factorial ANOVA with repeated measures on time were used to determine statistical differences with significance set at *p* ≤ 0.05.

## Results

There were no significant interaction, group, or time effects observed following six weeks of supplementation for nitrite (µmol·L^-1^) (ECH Pre: 0.88 ± 0.07 vs. ECH Post-42: 0.73 ± 0.10; PLA Pre: 0.91 ± 0.16 vs. PLA Post-42: 0.96 ± 0.22), nitrate (µmol·L^-1^) (ECH Pre: 17.44 ± 1.85 vs. ECH Post-42: 20.16 ± 2.23; PLA Pre: 16.01 ± 1.50 vs. PLA Post-42: 14.77 ± 1.21), or nitrite/nitrate (µmol·L^-1^) (ECH Pre: 18.32 ± 1.86 vs. ECH Post-42: 20.89 ± 2.25; PLA Pre: 16.92 ± 1.49 vs. PLA Post-42: 15.73 ± 1.22) or for any of the intermediate (day-14, -28) measurement points.

## Conclusions

These results suggest that six weeks of oral *Echinacea purpurea* supplementation (8,000 mg·d^-1^) did not significantly change nitrite, nitrate, or nitrite/nitrate. Therefore, *Echinacea purpurea* may not be an effective herbal supplement for enhancing NO production in apparently healthy, recreationally active, males with above average aerobic fitness (VO_2max_ = 52.9 ± 0.9 mL·kg^-1^·min^-1^).

